# Prune Belly Syndrome Associated with Interstitial 17q12 Microdeletion

**DOI:** 10.1155/2022/7364286

**Published:** 2022-02-14

**Authors:** Surasak Puvabanditsin, Miry Shim, Jeffrey Suell, Jeffrey Manzano, Kristin Blackledge, Avram Bursky-Tammam, Rajeev Mehta

**Affiliations:** Department of Pediatrics, Rutgers Robert Wood Johnson Medical School, New Brunswick, New Jersey, USA

## Abstract

We report a term male neonate presenting with a “prune belly,” bilateral hydronephrosis, hydroureter, posterior urethral obstruction, and bilateral undescended testes. Analysis with the whole genome SNP microarray revealed an interstitial deletion of about 1.49 megabase (MB) at chromosome 17q12. We present a rare association of prune belly syndrome with a chromosomal deletion in this same region.

## 1. Introduction

Interstitial deletions involving terminal 17q12 (17q12-qterm) are rare chromosomal abnormalities associated with variable phenotypes. Previous case reports of 17q12 microdeletion characterized the patients with structural and/or functional disorders of the kidney and urinary tract, maturity-onset diabetes of the young type 5 (MODY5), and neurodevelopmental or neuropsychiatric disorders (e.g., developmental delay, intellectual disability, autism spectrum disorder, schizophrenia, anxiety, and bipolar disorder) [1–3]. To date, there are few reported cases of prune belly syndrome (PBS) and associated chromosomal abnormalities. We report a new case of PBS associated with 17q12 microdeletion, and we review the literature.

## 2. Case Report

A term Hispanic male neonate was born at 40-week gestation to an 18-year-old primigravida by cesarean section. Apgar scores were 9 and 9 at 1 and 5 minutes, respectively. Pregnancy was complicated by an abnormal prenatal ultrasound at 20-week gestation which revealed bilateral renal pelvis dilation, dilated ureters, thickened bladder with keyhole sign, and dilated urethra. Subsequent sonography revealed progression of the hydronephrosis and hydroureters. Genetic studies that were performed during the pregnancy had shown 17q12 microdeletion, a de novo mutation. The family history was negative for congenital anomalies, and there were no history of in utero exposure to any known teratogens and no history of consanguinity.

**Figure 1:**
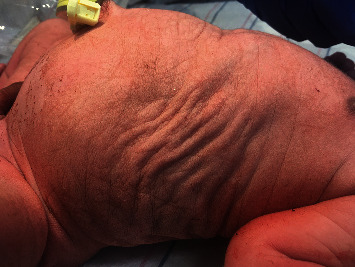
Note the wrinkled, flaccid, and thin abdominal wall.

**Figure 2:**
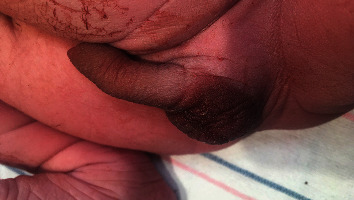
Note few rugae and undescended testes.

**Figure 3:**
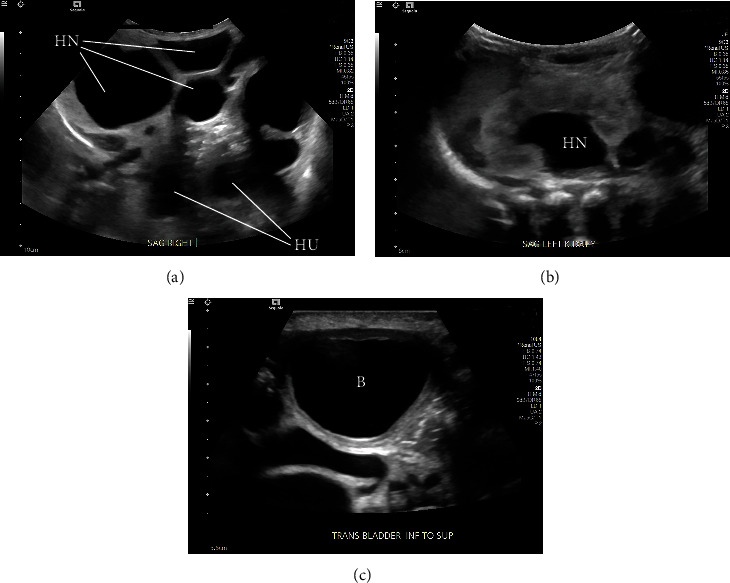
Renal and bladder sonography showed a severe (a) right hydroureteronephrosis, (b) moderate left hydroureteronephrosis, and (c) thick wall urinary bladder. HN: hydronephrosis; HU: hydroureter; B: urinary bladder.

**Figure 4:**
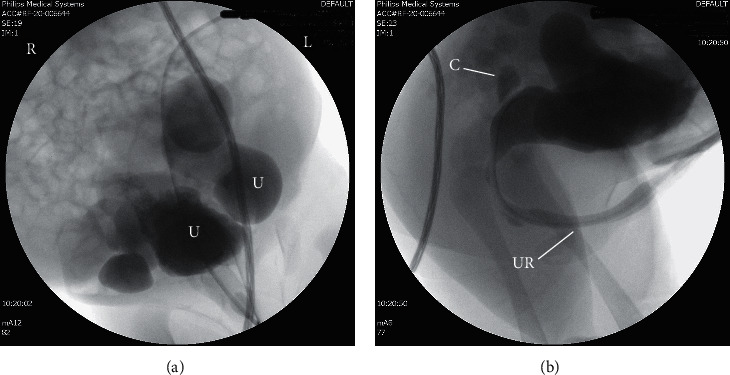
(a) Voiding cystourethrogram showed grade 5 left vesicoureteral reflux (ureter (U)). (b) Dilatation of the posterior and anterior urethra and cystic structure posterior to the urinary bladder (utricle cyst) (cyst (C), urethra (UR)).

Physical examination revealed a weight of 3470 grams (45^th^ centile), length of 50 cm (40^th^ centile), and head circumference of 35 cm (45^th^ centile). Anomalies noted at birth included marked abdominal distension with a thin, wrinkled, and flaccid abdominal wall and bilateral undescended testes (shown in Figures 1 and 2). A Foley catheter was placed on day of life (DOL) 0, and the infant was started on amoxicillin for urinary tract infection (UTI) prophylaxis. Ultrasound of the kidneys and bladder on DOL 0 showed severe right hydroureteronephrosis, moderate left hydroureteronephrosis, thick-walled urinary bladder, and dilatation of the posterior urethra. There was a cystic structure noted in the prostate (shown in Figure 3). An abdominal ultrasound found bilateral testes in the midabdomen. Voiding cystourethrogram (VCUG) performed on DOL 2 revealed grade 5 left vesicoureteral reflux and grade 1 right vesicoureteral reflux. The cystic structure in the prostate represented the prostatic utricle cyst on VCUG. The posterior urethral dilatation was noted which was probably the result of prostatic hypoplasia. The anterior urethral dilatation was also noted secondary to anterior urethral valve (shown in Figure 4). A MAG-3 renogram with Lasix on DOL 6 revealed split function of 43% in the left kidney and 57% in the right kidney without significant clearance after Lasix administration, which was consistent with bilateral obstruction. Echocardiogram showed a small patent ductus arteriosus and patent foramen ovale, and a neurosonogram was normal. The serum creatinine was monitored daily for the first few days of life and then twice weekly, peaking at 1.1 mg/dL on DOL 2 and 8.

Right and left nephrostomy tubes were placed on DOL 8 and 15, respectively. After the procedures, he developed *Enterobacter cloacae* pyelonephritis despite UTI prophylaxis. Upon discharge on DOL 21, serum creatinine was 0.8 mg/dL. The infant's feeds consisted of breastmilk or Similac PM 60/40. Bilateral nephrostomy tubes remained in places upon discharge; he was prescribed daily oral amoxicillin for the prevention of urinary tract infection. He was discharged to a subacute hospital for continued management of nephrostomy tubes until planned bilateral pyelostomy and orchiopexy at 1 month.

## 3. Cytogenetic and Molecular Studies

Whole genome SNP (single-nucleotide polymorphism) microarray analysis was performed using the SNP oligonucleotide microarray analysis (SOMA) CytoScan HD platform which uses over 743,000SCN probes and 1,953,000 NPCN probes with median spacing of 0.88 kilobase (kb). Total genomic DNA was extracted from the patient's blood sample and digested with NspI and ligated to NspI adaptors. Polymerase chain reaction (PCR) products were purified and quantified. Purified DNA was fragmented and biotin labeled and hybridized to the CytoScan HD GeneChip. There was a 1.49 megabase (MB) interstitial deletion of the long arm of chromosome 17: arr [hg19] 17q22 (34,822, 465-36, 307,773) x1 and 826 kilobase (kb) interstitial duplication in the long arm of chromosome 7: 7q12.1 (98,230,688-99, 056,822) x3.

The SNP microarray analysis identified an interstitial deletion of 17q22.1, and this deleted interval includes numerous Online Mendelian Inheritance in Man (OMIM) genes starting from *ZNHIT3* to *TBC1D3H*. Deletion of this region has been reported to be associated with the following phenotypes: cystic renal disorders, pancreatic atrophy, liver abnormalities, cognitive impairment and structural brain abnormalities, maturity-onset diabetes of the young (MODY), Müllerian aplasia/Mayer-Rokitansky-Küster-Hauser syndrome in females, and epilepsy (OMIM 189907).

Whole genome SNP microarray (Reveal) analysis identified an interstitial duplication of 7q12.1; this interval includes 10 OMIM genes (NPTX2, TRRAP, SMURF1, KPNA7, ARPC1A, ARCP1B, PDAP1, BUD31, PTCD1, and CPSF4). There has been no report of clinically established disorders with duplication of this region.

## 4. Discussion

Aplasia of the abdominal wall muscle was described by Froehlich in 1939, which is considered the first description of PBS [1]. The incidence of PBS is estimated about 1/30,000 to 1/50,000 live births [2–4], with the incidence decreasing likely due to improvement of prenatal detection and elective termination of affected pregnancies. Most of the cases of PBS are male (3-5% being female), prevalence is higher in the black population, and incidence is higher in infants born to younger mothers [5, 6]. The triad of PBS manifestation included (1) dysgenesis and partial or complete aplasia of muscle of the abdominal wall, (2) undescended testes, and (3) complex malformations of the urinary tract [7].

There are other findings apart from the triad, which are present in 75% of the PBS cases. Pulmonary abnormalities, e.g., pulmonary hypoplasia (not parenchymal lung disease), are present and related to prenatal oligohydramnios. Gastrointestinal complications included malrotation, intestinal atresia/stenosis, volvulus, and obstruction occurring in about 30% of PBS patients [8]. 10% of PBS cases have associated cardiac anomalies: septal defects, tetralogy of Fallot, and patent ductus arteriosus [3]. Musculoskeletal abnormalities, usually lower extremities: club feet, congenital hip dislocation, and hypoplasia of the leg or foot, were seen in 5% of the PBS cases [2, 9].

The pathogenesis of PBS has been debated and currently proposed with two prominent thoughts: mesenchymal developmental failure or urinary tract obstruction [10]. Higher incidence in males and cases of familial PBS lead to the speculation of a possible sex-influenced inheritance pattern. Murray et al. and Haeri et al. reported 2 cases of PBS with interstitial deletions of chromosome 17q12, suggesting the role of *HNF1B* (*TCF2*), a gene that is responsible for mesodermal and endodermal development in different tissues, in PBS [11–13]. However, 17q12 deletion has also been reported in association with other congenital abnormalities of the kidney and urinary tract (CAKUT) without the PBS phenotype: agenesis, hypoplasia, dysplasia, multicystic dysplastic kidney (MCDK), and horseshoe kidney, collecting system abnormalities (duplicated collecting systems, ureteropelvic junction obstruction, isolated hydronephrosis, or hydroureter) and tubulointerstitial disease of the kidney [14–18].

## 5. Conclusion

We report a case of a neonate with 17q12 microdeletion that is associated with PBS. Our report supports the genetic basis in PBS, and the screening for HNF1B gene mutations/deletions on chromosome 17q12 could help identify the patient of PBS and lead to preventing and improving the treatment of this rare disease.

## Data Availability

All data pertaining to this article are available from the authors.
